# How do crate materials impact the winter transport of broilers?

**DOI:** 10.5713/ab.24.0344

**Published:** 2024-10-28

**Authors:** Myunghwan Yu, Elijah Ogola Oketch, Nuwan Chamara Chathuranga, Shan Randima Nawarathne, Venuste Maniraguha, Bernadette Gerpacio. Sta. Cruz, Eunsoo Seo, Jeseok Lee, Jung Min Heo

**Affiliations:** 1 Department of Animal Science and Biotechnology, Chungnam National University, Daejeon 34134, Korea

**Keywords:** Animal Welfare, Broilers, Crate, Meat Quality, Stress, Transportation

## Abstract

**Objective:**

Pre-slaughter transportation adversely impacts the welfare, meat yield, and quality of broilers, yet the effects of different crate types on broiler chickens during winter remain underexplored. The goal of this study was to investigate the effect of plastic and iron crates in transit on meat quality, carcass, and physiological traits of broiler chickens during winter.

**Methods:**

A total of 175 (35-day-old) Ross 308 male broilers with an average body weight of about 1,708±33.3 g (mean±standard error of the mean) were picked after 4 hours of feed withdrawal before transport. The control group comprises birds in the farm (n = 15) without transportation at 173 cm^2^/kg density. The birds were transported into fixed iron (25 birds per crate) and plastic crates (15 birds per crate) with four replicates per crate type at the same 173 cm^2^/kg densities. The transportation distance was 20 km for 40 min at an average speed of 30–50 km/h early morning at 8:00 am under −1°C and 47% relative humidity.

**Results:**

There was no difference (p>0.05) in carcass traits among the treatments. Concerning meat quality, broilers transported in both crate types exhibited lower (p<0.01) a* values compared to the control group. Additionally, the iron crate group demonstrated higher (p<0.05) b* values for the breast meat compared to the other groups. In terms of blood metabolites, the iron crate group had higher (p<0.05) cortisol, glucose, and lactate levels compared to the control group that did not transport.

**Conclusion:**

Broilers transported in the iron crates increase stress levels in terms of higher cortisol, glucose, and lactate contents in the blood plasma compared to untransported broilers during the winter. Therefore, employing plastic crates, which induce significantly reduced cortisol and numerically lower glucose levels compared to iron crates, appears more favorable for animal welfare by mitigating stress.

## INTRODUCTION

The nutritional profile of poultry meat, including chicken, duck, and goose, aligns with the criteria of a modern healthy diet, which emphasizes high protein content, lower-calorie, -fat, -cholesterol levels, and efficient digestive absorption [1]. As a result of these benefits, global broiler meat production has increased significantly, reaching approximately 121.5 million metric tons, a 107% rise between 2000 and 2021, accompanied by a corresponding growth in consumption and demand [2,3]. In the process of poultry meat production, transportation of poultry from the farm to the slaughterhouse is an essential step and is recognized as a pivotal point in the production chain, with important implications for the welfare [4,5]. Furthermore, transportation is a critical stage characterized by various stressors including pre-transport handling, prolonged deprivation of feed and water, social disruption, exposure to noise, overcrowding movement, vehicle vibration, and exposure to extreme temperatures [2,6]. These stressors can lead to significant levels of stress, resulting in physiological and behavioral alterations such as heightened fear responses and economic losses for producers [7,8]. The stress induced during transportation affects the carcass yield and the muscles, impacting carcass quality during slaughter by depleting muscle glycogen reserves, altering muscle pH, and subsequently affecting storage conditions to a considerable degree [9,10].

Hence, the poultry sector is actively seeking methods to alleviate the stress associated with transportation through various interventions such as feed addition supplementation before transportation, optimizing transportation vehicle conditions, and implementing microclimate temperature control measures, aiming to ensure the welfare of poultry during transit [11–15]. However, there are few outcomes available about the effect that the type of crate can have on the welfare of the birds transported. The harvesting procedures and types of crates for transportation are various such as loose crates, fixed crates, and several modules [16]. In Korea, most of the transportation for broilers has now largely been used by loose crates as plastic crates, and fixed crate systems as iron crates [17].

The variety of crate materials used for transporting broilers varies worldwide due to factors such as operational size, available capital, technological advancements, economic viability, durability, and environmental concerns. Plastic crates are frequently favored globally for industry and experiments due to their convenience and ecological friendliness [5,18–20]. However, considerations such as environmental impact, hygiene standards, animal welfare, and regulatory requirements are becoming increasingly influential in crate material selection. Yu et al [21] examined the effects of using iron and plastic crates during summer transportation on the production, physiological traits, and welfare of broiler chickens. However, scarce information is available up to date regarding the effects of these materials on winter transportation. Thus, the objective of the present experiment was to investigate the effect of plastic and iron crates in transit on carcass, physiological traits, and meat quality in broiler chickens. The findings aim to provide valuable insights for improving broiler transportation practices, especially during challenging conditions like winter.

## MATERIALS AND METHODS

The Animal Ethics Committee of Chungnam National University, Daejeon, Republic of Korea, approved the protocols used in this experiment (approval number: 202212A-CNU-215).

### Birds, experimental design, and treatments

Before transportation, all birds were housed in Chungnam National University experiment farm which had 48 battery cages (76×61×46 cm^3^) that housed six birds until transportation and were managed according to the Ross 308 broiler management guideline [22]. A total of 175 Ross 308 male broilers, age 35 days with an average body weight of about 1,708±33.33 g (mean±standard error of the mean) in winter, were picked only healthy chickens weighing between 1.6 and 1.8 kg after 4 h of feed withdrawal before catching. To ensure consistent handling and minimize overall stress for the birds during the catching process, the Japanese method was followed [23]. Trained teams caught the birds from the cages and transported them securely, using both hands to hold their wings close to the handler’s body. The birds were transported in different types of crates as follows: an iron crate having dimensions of 1.00 m (length)×0.78 m (width)×0.26 m (height); a plastic crate (ECO FARM, Jincheon, Korea) having dimensions of 0.82 m (length)×0.57 m (width)×0.29 m (height) with 4 replicates per crate type. The iron crates were custom-made from transport vehicles, whereas the plastic crates used were the standard models typically used for broiler transport in Korea. The control group comprises birds in the farm (n = 15) without crating and transportation while applying four hours of feed withdrawal. The ambient temperature for the control group was maintained at 24±1°C in the experiment according to the Ross 308 broiler management guidelines [22]. The birds were placed in crates based on optimal crating density suggested by the Korean transportation standards [24] of an average of 173 cm^2^/kg, and the control group was also comprised of the same density. The transportation’s distance was 20 km for 40 min at an average speed of 30 to 50 km/h during the early morning from 8:00 am. The weather was bright and sunny, the average temperature was −1°C, and the relative humidity was 47%. Temperature and humidity data were obtained from the Meteorological Agency records.

### Sample collection and carcass traits

Eight birds per group were selected based on closeness to the mean body weight of the birds in the respective crate and cage (i.e., two birds per crate and cage) after transportation was completed. Subsequently, their weights were recorded as the live body weight. Blood samples were collected from the brachial vein into a vacutainer coated with lithium heparin (BD Vacutainer; BD, Franklin Lakes, NJ, USA) before euthanizing the birds. The birds were then euthanized using carbon dioxide asphyxiation for the evaluation of some carcass characteristics. The dressing percentage with giblets (i.e., heart, gizzard, and liver) was determined as a function of the live weight of the birds. The breast and leg meats were then separated and weighed to measure them relative to the total carcass weight. The breast meat of broilers was then collected for meat quality analyses [25].

### Physiological responses

Collected blood samples were centrifuged (LABOGENE 1248R; Gyrozen, Daejeon, Korea) at 3,000×g for 10 min at 4°C and the plasma was separated and stored at −80°C (UniFreez U 400; DAIHAN Scientific, Wonju, Korea) until analysis. Corticosterone levels were measured from the plasma using a corticosterone enzyme-linked immunosorbent assay (ELISA) kit (CSB-E11991C; CUSABIO, Wuhan, China) according to the manufacturer’s guidelines. Plasma cortisol concentrations were assessed with a cortisol ELISA kit (CSB-EQ027342CH; CUSABIO) following the manufacturer’s instructions. Lactate concentration was determined by lactate assay kit (MAK065; Sigma Aldrich, Burlington, MA, USA) using the manufacturer’s instructions. Additionally, plasma glucose levels were determined using a glucose assay kit (AM201-K; Asan Pharmaceutical Co., Ltd., Seoul, Korea), in accordance with the manufacturer’s instructions [26].

### Physicochemical traits

The pH values of the breast meat were monitored immediately after sample collection. An aliquot (9 mL) of distilled water was added to 1 g of muscle, followed by homogenization (T25 basis; IKA-Werke GmbH & Co. KG, Staufen im Breisgau, Germany) for 30 seconds. The homogenate was centrifuged at 2,090×g (ScanSpeed 1580R; Labogene ApS, Lillerød, Denmark) for 10 min and the supernatant was filtered through filter paper (No. 4; Whatman, Maidstone, UK). The pH of the filtrate was measured using a pH meter (SevenEasy; Mettler-Toledo Intl. Inc., Schwerzenbach, Switzerland).

The Commission Internationale de l’Eclairage (CIE) lightness (L*), redness (a*), and yellowness (b*) of broiler breast meat were determined using a spectrophotometer (CM-3500d; Minolta Inc., Tokyo, Japan). Measurements were taken perpendicularly to the surface of the broiler breast meat with a 30 mm diameter of illumination area at two different locations per sample. The results were analyzed in the SpectraMagic software (SpectramagicTM NX; Konica Minolta Inc.).

For the water holding capacity (WHC) measurements, the 2 g of raw broiler breast meat sample weighed exactly was placed on cotton wool, and added to a centrifuge tube. The weight of the meat after centrifugation at 2,090×g (ScanSpeed 1580R; Labogene ApS) for 10 min was measured and compared to the initial meat weight. The moisture content of meat was determined by drying 2 g of samples placed in aluminum dishes for 3 h at 110°C. The remaining moisture (%) present in the meat after centrifugation was expressed as the WHC [27].

To measure the cooking loss, the breast meat of the broiler was weighed vacuum packaged, and cooked for 20 min in a water bath at 80°C until the internal temperature reached 70°C. The cooked breast meat of broilers was cooled at room temperature (20°C) for 30 min. After the vacuum bag was removed, the surface moisture of the breast meat of the broiler was removed with paper towels, and the cooked breast meat of the broiler was weighed. The cooking loss was calculated as the difference between the weight of raw breast meat and cooked breast meat.

### Statistical analysis

The data obtained from the experiment were analyzed by one-way analysis of variance (ANOVA) in SPSS (IBM SPSS Version 26; SPSS Inc., Chicago, IL, USA). For carcass traits, meat quality, and blood metabolites, selected individual birds from each crate (8 per treatment) were considered as the experimental unit. Statistical significance was determined at a significance level of p<0.05. Also, trends (tendencies for significant effects) were measured at 0.05<p<0.10. Significant treatment effects were separated using Tukey’s multiple-range test.

## RESULTS AND DISCUSSION

The broilers used in the experiment were transported according to the appropriate transport density specified in Korean Law [24], and subsequent mortality never occurred in the current study.

### Carcass traits

The carcass traits represent a vital economic component within the broiler industry [28]. The data from Table 1 showed, there was no difference (p>0.05) in carcass traits among the treatments. Transportation-induced stress leads to weight reduction (p<0.05), elevated rates of injury, diminished production performance, and, in extreme instances, can result in considerable mortality [29]. While previous studies [6,13,19,26] examining transportation factors such as crate density, duration, and distance affected variations in carcass traits, our present study indicated that the crate types had no discernible impact on carcass yield. Hussnain et al [19] found that broilers transported in 240 km exhibited lower carcass yield and breast meat percentage, along with higher serum catalase levels, compared to those transported in 80 km during the winter. Catalase, an antioxidant enzyme, serves as an indicator of oxidative stress in living organisms [30]. This suggests that longer transport distances increase stress levels in broilers. Thus, the variability in outcomes could be attributed to insufficient stress during short transportation periods, which influences carcass characteristics. Additionally, it could also be linked to the varied experimental designs and environments utilized in each respective study. Moreover, the birds had adequate space allowance for transportation, which could not affect the carcass characteristics, although we found somewhat higher stressor indices. Furthermore,

Dos Santos et al [5] observed that the interaction of season (rainy and dry) and transportation distance (90 km and 15 km) did not significantly impact the prevalence of bruising on carcasses. Likewise, Hussnain et al [13] and Hussnain et al [19] demonstrated that neither transportation distance nor crate density, nor their interaction, had a noticeable effect on physical injuries or bruising in the breast and wing areas. Although physical injuries were not examined in our study, further research is necessary to determine the economic feasibility of meat production.

### Physiological responses

**Figure 1:**
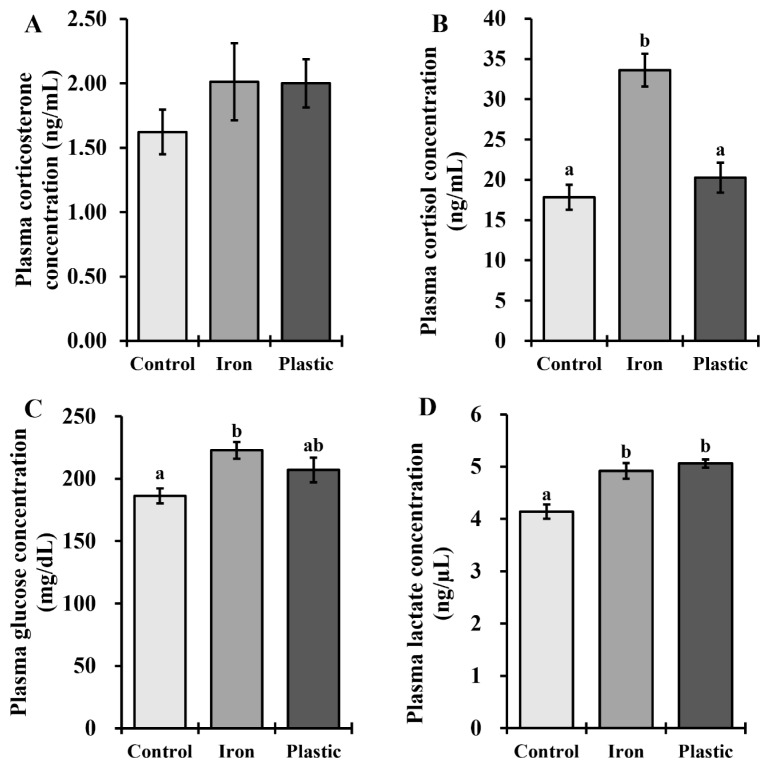
Blood metabolites of broilers under different materials of crates during transportation in winter. The (A) plasma corticosterone concentration, (B) plasma cortisol concentration, (C) plasma glucose concentration, and (D) plasma lactate concentration are presented. The values in the histogram are the means±standard errors of the mean (n = 8). ^a,b^ Values with different letters are significantly different (p<0.05).

The results of the blood metabolites of the broiler using the types of crates during transportation are presented in Figure 1. Broilers transported in iron crates had (p<0.05) increased cortisol, glucose, and lactate contents compared with the control group that did not transport. Transportation stress on broiler chickens was evident here, as the plasma contents of cortisol, glucose, and lactate increased. Corticosterone and cortisol serve as indicators of biological stress and can be detected in a range of species, including poultry and swine [6,31]. Gou et al [6] and Yu et al [32] observed that higher transport crating density and longer transport distances resulted in elevated levels of both cortisol and corticosterone. In this present study, we observed that broilers transported with iron crates exhibited significantly elevated cortisol levels when compared to those transported in plastic crates, and the control group. Iron is characterized by its significant heat capacity and excellent thermal conductivity when used structurally [33]. Conversely, plastic materials provide effective protection against weather conditions like heat, cold, rain, and snow owing to their inherent properties of low thermal conductivity and resistance to moisture [34]. These differences in crate materials suggested that the elevated plasma cortisol levels induced by cold stress could be attributed to the iron crate’s higher heat capacity and thermal conductivity compared to the plastic crate in cold climates. In this regard, broilers transported in iron crates might suffer more cold stress due to feeling cold than those transported in plastic crates. Similarly, broilers transported in iron crates observed higher (p<0.05) glucose levels than the control group. Glucose acts as an easily accessible energy source for cellular functions and is also recognized as an indicator of stress [35]. It has been observed that transportation stress leads to an increase in plasma glucose levels, primarily attributed to the breakdown of glycogen in the liver [19,26,36]. Despite the same transportation procedures, the difference in glucose levels between the plastic and iron crates is believed to stem from the degrees of transport stress induced by the differing materials, as discussed earlier. During periods of stress, animals undergo a metabolic pathway wherein glycogen, glucose, and glucose-6-phosphate are converted into lactate. The accumulation of lactate leads to a decrease in overall pH levels, subsequently impacting the color and WHC of meat [26,36]. In the current study, broilers transported in both types of crates displayed increased lactate levels in comparison to the control group. We propose that this rise can be attributed to the struggling activity and exposure to cold stress experienced by broilers during transportation.

### Physicochemical traits

Table 2 shows the effect of crate material types for transportation on physicochemical traits. No notable differences (p>0.05) were observed among the treatments regarding pH, WHC, cooking loss, and L* values of breast meat. However, broilers transported in both crate types showed lower (p<0.01) a* values compared to the control group. Additionally, the iron crate group demonstrated higher (p<0.05) b* values for breast meat compared to the other groups. Meat color is one of the most influential visual appearance traits, as it often serves as the primary factor for consumers when choosing meat at the point of purchase [37]. The red value (a*) of meat is typically influenced by pigment state and structure. Fresh muscle color, which consumers prefer, is largely due to red oxygenated myoglobin, which can reversibly convert to brown metmyoglobin and is affected by temperature, pH, and oxidative conditions during transport [29]. In this study, the a* value was higher in the transported groups compared to the control group. Given the lack of significant changes in muscle pH, this increase is likely due to cellular oxidative stress resulting from transport conditions. The potential of hydrogen (pH) is crucial for assessing chicken meat quality as it is influenced by the glycogen levels in muscles before slaughter and the conversion rate of glycogen to lactic acid after slaughter. In this current study, the lactate levels were significantly higher in the transported groups, regardless of the type of crate, than in the control group. A reduced pH level was also observed in the transported group, regardless of the crate type, compared to the control group. This suggests that lactate accumulation due to transport stress led to lower pH levels in broiler breast meat [36]. Contradictory, Yu et al [21] reported that broilers transported in plastic crates had lower plasma lactate levels compared to those in iron crates, with no significant pH difference between the crate types during summer. These differing results are likely due to varying stress levels across changing seasons. Lower pH levels are linked to diminished WHC in meat, resulting in higher cooking loss, drip loss, and reduced tenderness [38,39]. Zhang et al [36] reported that the ultimate pH levels and drip loss of breast meat in broilers remained consistent, irrespective of transportation, aligning with our experimental findings. Similarly, Gou et al [6] noted that despite the increasing transportation duration, there were no significant alterations in the ultimate pH and color of breast meat in Yellow broiler chickens. However, it observed a rise in drip loss with longer transport durations. Furthermore, Debut et al [40] found no disparities in breast meat color between transported and non-transported broiler chickens. In this light, variations in transportation conditions, such as weather, distance traveled, duration of transport, and the genetic nature of the broilers, are considered the primary factors contributing to the inconsistencies found in studies exploring the influence of transportation on meat quality.

## CONCLUSION

In conclusion, the transportation of broilers to iron crates can increase stress levels in terms of significantly higher cortisol, glucose, and lactate contents in the blood plasma than untransported broilers in the winter. Consequently, using plastic crates, which resulted in notably reduced cortisol levels and numerically lower glucose levels in comparison to iron crates, seems to be a preferable option for promoting animal welfare by reducing stress.

## Figures and Tables

**Table 1:** Carcass traits as affected by different materials of crates during transportation in winter

Items (%)	Control	Iron	plastic	SEM^1)^	p-value
Dressing ratio^2)^	88.80	88.36	88.87	0.239	0.648
Relative breast meat weight^3)^	30.65	30.91	31.08	0.440	0.924
Relative drumstick weight^4)^	5.31	5.85	5.34	0.097	0.068

1) Pooled standard error of mean.

2) (Carcass weight/live body weight)×100

3) (Breast meat weight/carcass weight)×100

4) (Drumstick weight/carcass weight)×100

**Table 2:** Physicochemical traits of chicken breast meat under different materials of crates during transportation in winter

Items	Control	Iron	Plastic	SEM^1)^	p-value
pH	6.04	5.89	5.96	0.097	0.068
WHC (%)	74.40	71.98	73.67	0.730	0.404
Cooking loss (%)	21.90	21.42	21.08	0.444	0.752
L*	50.76	51.26	50.84	0.433	0.877
a*	9.02^b^	7.85^a^	8.10^a^	0.150	0.008
b*	18.22^a^	20.07^b^	18.52^a^	0.249	0.010

WHC, water holding capacity.

1) Pooled standard error of mean.

a,b Values in a row with different superscripts differ significantly (p<0.05).
